# Effects of Increased Nitrogen Deposition and Rotation Length on Long-Term Productivity of *Cunninghamia lanceolata* Plantation in Southern China

**DOI:** 10.1371/journal.pone.0055376

**Published:** 2013-02-04

**Authors:** Meifang Zhao, Wenhua Xiang, Dalun Tian, Xiangwen Deng, Zhihong Huang, Xiaolu Zhou, Changhui Peng

**Affiliations:** 1 Faculty of Life Science and Technology, Central South University of Forestry and Technology, Changsha, Hunan, People’s Republic of China; 2 Institute of Environment Sciences, Department of Biological Sciences, University of Quebec at Montreal, Montreal, Quebec, Canada; Cirad, France

## Abstract

*Cunninghamia lanceolata* (Lamb.) Hook. has been widely planted in subtropical China to meet increasing timber demands, leading to short-rotation practices that deplete soil nutrients. However, increased nitrogen (N) deposition offsets soil N depletion. While long-term experimental data investigating the coupled effects related to short rotation practices and increasing N deposition are scarce, applying model simulations may yield insights. In this study, the CenW3.1 model was validated and parameterized using data from pure *C*. *lanceolata* plantations. The model was then used to simulate various changes in long-term productivity. Results indicated that responses of productivity of *C*. *lanceolata* plantation to increased N deposition were more related to stand age than N addition, depending on the proportion and age of growing forests. Our results have also shown a rapid peak in growth and N dynamics. The peak is reached sooner and is higher under higher level of N deposition. Short rotation lengths had a greater effect on productivity and N dynamics than high N deposition levels. Productivity and N dynamics decreased as the rotation length decreased. Total productivity levels suggest that a 30-year rotation length maximizes productivity at the 4.9 kg N ha^−1^ year^−1^ deposition level. For a specific rotation length, higher N deposition levels resulted in greater overall ecosystem C and N storage, but this positive correlation tendency gradually slowed down with increasing N deposition levels. More pronounced differences in N deposition levels occurred as rotation length decreased. To sustain *C*. *lanceolata* plantation productivity without offsite detrimental N effects, the appropriate rotation length is about 20–30 years for N deposition levels below 50 kg N ha^−1^ year^−1^ and about 15–20 years for N deposition levels above 50 kg N ha^−1^ year^−1^. These results highlight the importance of assessing N effects on carbon management and the long-term productivity of forest ecosystems.

## Introduction

Nitrogen (N) is the element that has the greatest limiting effect on plantation productivity [1]. However, in many areas, forest ecosystems have experienced increased atmospheric N deposition in recent years [2]–[5]. For example, in most forests in subtropical China, the current rate of N deposition ranges from 18 to 73 kg N ha^−1^ year^−1^, and this is expected to increase in coming decades [6]–[9]. The increase in N that is deposited in forests will increase soil N content and, consequently, stimulate increased productivity over the short term [10]. Although N deposition could meet the N requirements of these forests, excess N will result in a nutrimental burden and offsite environmental effects [5], [11], [12]. Balancing the N supply and the demand for tree growth in these forests is critical for optimizing productivity, sustainability, and environmental protection [13], [14].

N cycling in forest ecosystems is a complex process because of the numerous soil–plant interactions. Nutrient pools and fluxes, as well as the nutrient requirements of the forest trees, vary as the stands develop [15]. To determine the effects of management practices on nutrient cycling and the responses in forest productivity that occur following changes in N input, long-term observational data sets are required. Previous studies have manipulated N levels to examine the short-term responses in tree growth and assess N requirements. However, these studies did not consider variations in the long term productivity and the N requirements of forest stands as they develop. Mechanistic models can accurately predict long-term changes in productivity and are widely used in N fertilizer applications, resulting in increased N-use efficiency and reduced pollution [15], [16]. Some mechanistic models have been used to analyze the effects of rotation length on tree and soil carbon (C) stocks and the quality of wood in different European forests [17], the effects of harvesting intensity and rotation length on long-term soil N and C dynamics in boreal forests in central Canada [18], the effects of N deposition, climate change, and rotation length on forest carbon sequestration and the harvesting of Scots pine forests in southern Finland [19], and to compare the ecological impact of natural disturbances and harvesting [20].

*Cunninghamia lanceolata* (Lamb.) Hook. is the third most commonly planted tree species in plantations in the world [21], and its total plantation area is now 9.21 million hectares in China [22]. Due to its high value in terms of timber quality and versatility, *C*. *lanceolata* has mainly been planted for timber production, but its bark and roots are usually harvested for local construction purposes and fuel [23], [24]. Currently, most *C*. *lanceolata* plantations are monocultures and successive rotation planting is commonly practiced [24], [25]. Increased timber demand and improvements in processing techniques have resulted in shorter harvesting cycles of *C*. *lanceolata* plantations (originally 30 years to now ≤20 years). Soil N depletion due to timber removal has been recognized as the major factor responsible for the decline in yields of *C*. *lanceolata* plantations [26]. Therefore, sustaining the productivity of *C*. *lanceolata* plantations over successive rotations is a concern of numerous researchers [27]–[29] and has been identified as a priority for plantation management in China [4]. Regarding *C*. *lanceolata* plantations, various models have been used to simulate age-related C dynamics [30], [31], yield decline due to management activities and degradation of soil fertility [28], and C sequestration at N deposition levels <1–50 kg N ha^−1^ year^−1^ at various rotation cycles [32]. However, the simultaneous effects of increased N deposition and rotation lengths with successive planting on long-term production and management in *C*. *lanceolata* plantations have not been studied so far.

This study uses the CenW3.1 model [33], [34] to simulate long-term production and N requirement dynamics in response to N deposition and different rotation lengths and establish sound management practices for the sustainable productivity of *C*. *lanceolata* plantations. The major parameters of production and N dynamics include stem wood biomass, net primary production (NPP), annual N requirement (ANR) for tree growth, soil available N for growth, and soil organic C (SOC). Specifically, the purposes of this study are to (1) determine how increased N deposition affects forest productivity as stand development; (2) examine the interactive effects of N deposition and rotation length on long-term forest productivity; (3) investigate whether practicing increased atmosphere N with shorter rotation that can avert N saturation damage in the study region.

## Materials and Methods

### Site Description

The *C*. *lanceolata* plantations that were evaluated in this study are located at the experimental area of the Huitong National Forest Ecosystem Research Station (NFERS) (lat 26°50′N, long 109°45′E), Hunan Province, southern China [31]. The region is characterized by a humid mid-subtropical monsoon climate. The annual mean temperature is 16.5°C, which ranges from an average of 4.3°C during the coolest month (January) to 29.4°C during the warmest month (July). The annual rainfall is approximately 1270–1650 mm, mainly occurring between April and August. The soil is a subgroup of clay loam red soil that is formed from shale and slate parent materials (Table 1).

**Table 1:** Soil characteristics of the *C*. *lanceolata* plantations at the Huitong National Forest Ecosystem Research Station, Hunan Province, China.

Characteristic	Value	Source
Soil pH	4.32–4.86	[60]
Bulk density (g cm^−3^)	1.09–1.7	[61]
Porosity (% of the total volume)	52.1–62.5	[62]
Permeable velocity (cc min^−1^)	2.83–10.1	[62]
Maximum moisture holding capacity (% of the total volume)	36.1–40.4	[62]
Moisture content (%) for most of the year	>30	[63]–[64]
Total N (g kg^−1^)	1.876–2.478	[65]
NH_4_^+^-N (mg kg^−1^)	6.67–9.1	[66]
NO_3_-N (mg kg^−1^)	11.2–24.3	[67]
Total P (g kg^−1^)	0.92–1.42	[68]
Total K (g kg^−1^)	4.27–5.13	[69]
TOC (g kg^−1^)	21.1–33.4	[70]
Soil C:N ratio	13–16	[64]

Eight *C*. *lanceolata* plantation watersheds were established within the research station in 1984. Automatic weather stations were installed within the plantations to collect climatic data. The first-rotation *C*. *lanceolata* plantations were established on a clear-cut of natural forest in 1966. After the slashed understory plants and harvesting residues had been burned, the soil was prepared by digging holes and 1-year-old *C*. *lanceolata* seedlings were planted. No weed control, fertilization or other treatment was employed. The establishment, survival, and growth of the plantation have been described previously [31]. This study was carried out at two watersheds (II and III), situated within NFERS. The second successive rotation plantation was established in watershed III in February 1987 and in watershed II in March 1995 with an initial planting density of 3318 trees ha^−1^ using identical planting methods as used in the first rotation. The ecological factors and characteristics of the *C*. *lanceolata* plantations related to this study were previously described [31]. The biomass and stand characters, including height (H, m), diameter at breast height (DBH, cm), and stand density (trees ha^−1^), were measured at different growing-stages beginning at age 7. Total volume (under bark) and dry matter biomass of the wood, bark, branches, and needles were calculated. Biomass, litter, forest floor, and understory samples were collected at an interval of five years for nutrient concentration and growth analysis. Linear regression was used to test the relationships between soil bulk density and soil C content.

### CenW Model

The CenW3.1 model was developed as a comprehensive monoculture forest model that runs in daily time-step with the C gain that is calculated from light absorption and can be modified by taking into account temperature, soil water status, and foliage N concentration [34]. CenW allows the explicit modeling of both C and N pools as well as their fluxes, thereby integrating tree growth parameters, environmental factors, and management treatments. The projection of stand growth and N dynamics are based on the rates of key ecological processes regulating the availability of and competition for light and nutrient resources. Net primary productivity (NPP, t ha^−1^ year^−1^, represents the remains of stand-level photosynthesis after subtracting losses of stand-level respiration and litterfall) can be reduced through a number of processes, such as increasing respiration, increasing senescence and mortality losses, immobilisation of nutrients and unfavourable shifts in biomass allocation. Fixed photosynthate is used for plant growth, with allocation to different plant organs determined by plant nutrient status, tree height and species-specific allocation factors. Stem wood biomass (t DM ha^−1^) includes heartwood and sapwood. Tree death is estimated as a simple daily fractional mortality rate. It is assumed that the ratio of above- to below-ground allocation increases with foliar N concentration. Foliar N concentrations are essentially determined through the relative rates of C and N uptake. It is also assumed that 25% of foliar N is relocated to other plant part before litter fall. Water use is calculated using the Penman-Monteith equation, with canopy resistance given by the inverse of stomatal conductance, which, in turn, is linked to calculated photosynthetic carbon gain. Water is lost through transpiration and soil evaporation, and gained by rainfall or irrigation which together determines soil water status for the following day. Stand density was fully independently modeled as a result of self-thinning. The nutrient cycle is considered through litter production by the shedding of plant parts, such as roots, bark, branches and foliage. Litter is assumed to be produced as a constant fraction of live biomass pools. In addition, foliage is shed during drought or when canopies become too dense. Litter is then added to the organic matter pools from where C is eventually lost and N becomes available again as inorganic mineral N. N can come from external addition (atmospheric deposition and fertilizer addition) or mineralization during the decomposition of soil organic matter. Plant available N depends on is current soil available N status plus additions from atmospheric deposition. Soil decomposition rate is determined by temperature, soil water status and soil organic matter quality. Input litter tends to have a wide C:N ratio so that some C needs to be lost through decomposition before critical C:N ratio is reached in organic matter and excess N can be mineralized. The model also offers the potential to manipulate forests in a variety of experimental ways and predict outputs. Detailed descriptions of model features, structures, mathematical representations, sensitivity analyses, and building strategies have been described [33], [34].

### Model Calibration and Validation

The calibration and validation of CenW3.1 in *C*. *lanceolata* plantations in this study followed two steps. The first step for model calibration was to select the data from watershed II to calibrate CenW3.1 model. The purpose of this step was to determine the model parameters to ensure simulation accuracy as much as possible. The second step for model validation was to evaluate the model against the independent watershed III to test the model accuracy. The data used in the model simulation included environmental factors, tree growth parameters, tree-N response data, forest floor decomposition rates, and N inputs. The primary parameters and values for productivity, above-ground biomass, decomposition, and soil C were previously described in detail [31] (see Appendix S1).

For this study, the CenW3.1 simulations were run for a period of 120 years using climate records that were obtained during the period from 1987 to 2010. The historic records of daily maximum and minimum values of air temperature, daily sum of radiation (MJ m^−2^ day^−1^), and daily precipitation (mm) over the period 1987–2010 at the study site were used for climate inputs. For future periods without observed climate data, we randomly assign each year’s climate condition with detrended climate data [35].

Tree growth parameters, tree-N response data, forest floor decomposition rates, and N inputs were obtained from the literature or field measurements (see Appendix S1). The rates of these processes were calculated from a combination of historical bioassay data (biomass accumulation in component pools, stand density) and measures decomposition rates and photosynthetic saturation curves by relating ‘biologically active’ biomass components (foliage and small roots) to calculate nutrient uptake, the capture of light energy, and net primary production. Then, the model generated a suite of growth properties for each tree components to be represented based on the historical data (i.e. growth and yield tables or long-term permanent plots), which was used to calibrate tree growth. These growth properties were subsequently used to simulate plant growth as a function of resource availability and management practices. Calibration data were assembled that describe the accumulation of tree biomass (above and below-ground components) as stands developed. Aging was taking into account by using calibration data pairs for different stand ages (i.e. age vs. tree size, age vs. stem density, age vs. biomass, etc.). Tree biomass and stand self-thinning rate data were estimated from the height, DBH and stand density as output of traditional empirical growth and yield models in conjunction with species-specific component biomass allometric equations. To calibrate the nutritional aspects of the model, data describing the concentration of nutrients in the various biomass components were required. The data required in the model on the degree of shading produced by different quantities of foliage and the response of foliage to different light levels were derived from literature values, field measurements, or simulation models. Lastly, decomposition rates of various litter types and soil organic matter were required for the model to simulate nutrient cycling. Fertilizer applications, irrigation management techniques, weather conditions (such as sunny, cloudy, and etc.), incidences of insect problems or disease, plowing, and fire were excluded in the simulations.

Moreover, the annual N requirement (ANR) for tree growth was defined as the year-to-year change in amounts of N required for biomass accumulated during the year (expressed in kg N ha^−1^ year^−1^). This was estimated using the following equation:
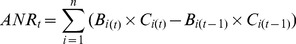
(1)where *ANR_t_* is the annual N requirement at stand age of year *t*; *B_i(t)_* and *B_i(t−_*_1*)*_ represent the accumulated biomass of tree compartment *i* at stand age of year *t* and *t−*1, respectively; *C_i(t)_* and *C_i(t−_*_1*)*_ represent the N concentration in tree compartment *i* at stand age of year *t* and *t−*1; and *n* is the number of compartments (including sapwood, heartwood, bark, branches, leaves, fine root, coarse root, flower, and fruits) within the stand. *B_i_* was quantified as the amount of biomass in each compartment *i*, plus the biomass returned to the soil as litter, predicted by CenW3.1 for each stand during each rotation cycle.

**Figure 1:**
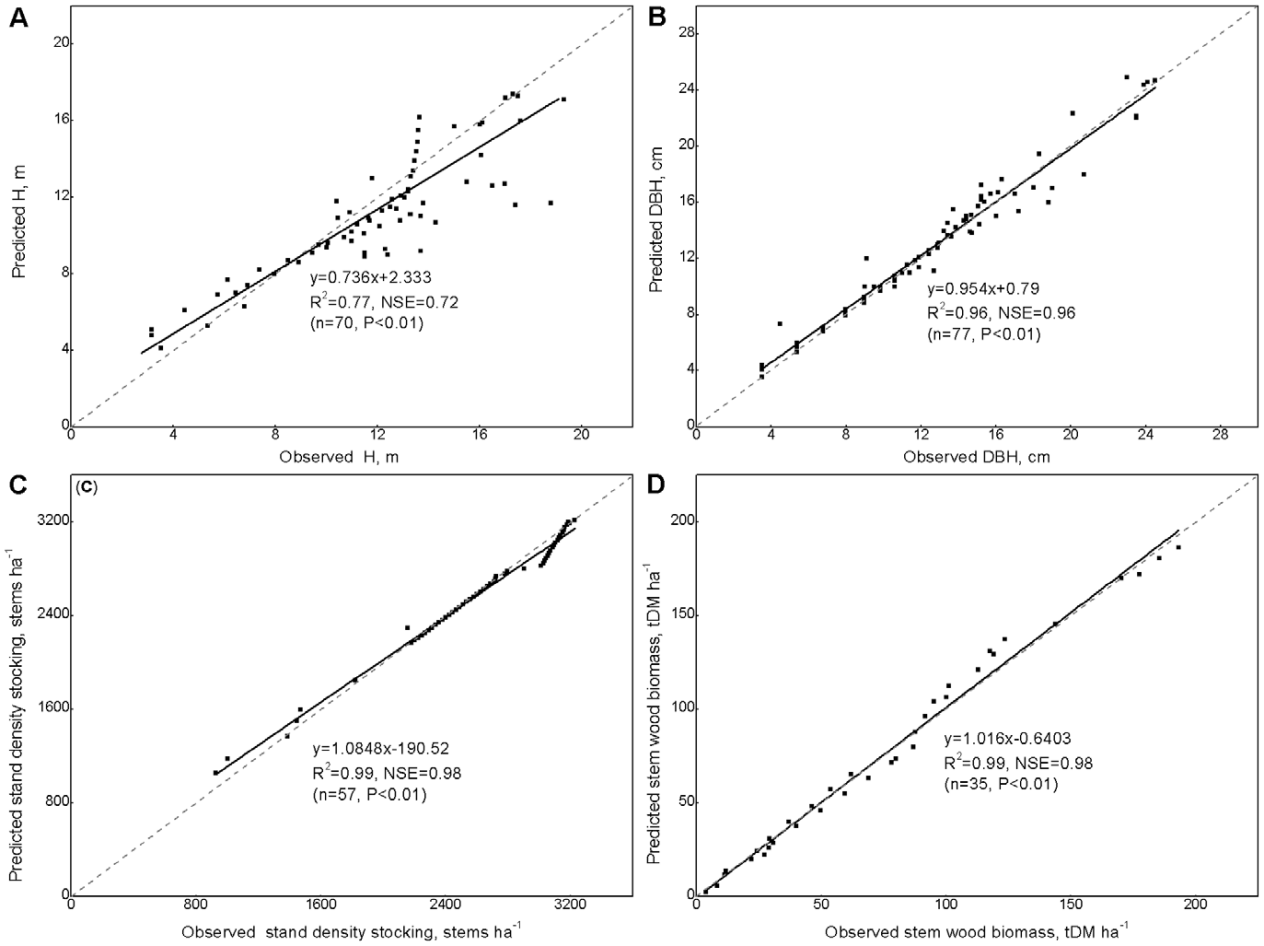
The model was validated by fitting the predicted and observed values. (A) Stand height (H, m). (B) Diameter at breast height (DBH, cm). (C) Stand stocking (stems per ha). (D) Stem woody biomass in tone dry matter per hector (t DM ha^−1^). Gray dashed lines represent a 1∶1 relationship; solid lines are linear regressions. NSE for “Nash-Sutcliffe efficiency”.

Linear regressions of the observed data obtained from the study site were compared with the simulated average stand height (H), average diameter at breast height (DBH), stand density (stems per hectare), and stem woody dry matter biomass that were calculated using CenW3.1. These comparisons were used to validate the CenW3.1 model for long-term predictions (Fig. 1). For a better quantification of the error in fitting low flows, the efficiency proposed by Nash and Sutcliffe (NSE) [36], defined as one minus the sum of the absolute squared differences between the predicted and observed values normalized by the variance of the observed values during the period under investigation, was used to tested modeling performances.

The H value (Fig. 1A) that was calculated by the model fitted well with the observations across different climatic conditions (R^2^ = 0.77, NSE = 0.72). The predicted and observed data were closely matched for DBH (Fig. 1B; R^2^ = 0.96, NSE = 0.96), stand density (Fig. 1C; R^2^ = 0.99, NSE = 0.98), and stem woody dry matter mass (Fig. 1D; R^2^ = 0.99, NSE = 0.98) in *C*. *lanceolata* plantations during the period 1990–2010. The validation results, therefore, confirm the reliability of the applied model for further investigations into the long-term effects of N deposition and rotation lengths on the productivity and N dynamics of *C*. *lanceolata* plantations.

### Model Simulation Runs

Five dynamic variables including stem wood biomass, NPP, ANR, soil available N for growth (kg N ha^−1^), and soil organic C (SOC, t C ha^−1^), were consider in this study. However, these variables are sometimes not sufficient to account for the full magnitude of changes. This study combines that response with simple assumptions about external N depositions and current rotation length practices. The atmospheric N depositions (kg N ha^−1^ year^−1^) refers to external input rates as wet and dry atmospheric deposition. For effects of increased N deposition on long-term forest productivity, five N deposition levels e.g. 4.9, 18, 30, 50, 70 and 90 kg N ha^−1^ year^−1^ were applied during the simulations. Based on the reported N depositions in China, the initial N deposition in 1987 is approximately estimated to be 4.9 kg N ha^−1^ year^−1^ (N4.9) at the study site [37], which represents the average deposition rate in areas with human population without industrial development. A level of 18 kg N ha^−1^ year^−1^ (N18) is the current deposition rate in southern China [8]. Whereas in the polluted areas in Hunan Province, levels around 30 kg N ha^−1^ year^−1^ (N30) is the most common, but reaches up to 50 kg N ha^−1^ year^−1^ (N50) [8]. Higher levels over 70 kg N ha^−1^ year^−1^ (N70) in the large industrialized urban areas have been reported. It could further increases up to 90 kg N ha^−1^ year^−1^ (N90) for near future [4]. In the model, it was taken to be a constant rate throughout the life of the stand evenly distributed into every day. Assuming no major climatic changes, the atmospheric N depositions alone is relatively constant for a given site over time. We will also assume that it is constant within a given rotation and over successive rotations. Harvesting and thinning affect C and N dynamics in different ways, depending on the harvesting intensity and the rotation length. In this study, C and N dynamics were simulated and compared using rotation lengths of 120 (RL120), 40 (RL40), 30 (RL30), 20 (RL20) and 15 (RL15) years over a period of 120 years. All rotations started with the planting of 3318 seedlings per hectare. At the end of each rotation period, the stands were clear-felled, and bole only harvesting was adopted, the stem wood at harvest was considered as removed from the site. When a bole harvesting only event occurs, 94% the wood and bark N and volume were removed from the site. The remaining 6% is transferred to the forest floor. The rate of removal of foliage and branch N and volume from the site remains at zero, since the entire amount is transferred to the forest floor as slash. The long-term growth was seldom affected by residue treatment and field observations generally confirm this [38].

The model was used to simulate growth (physiological) constrains on long-term productivity in the study site over a period of 120 years without the effects of rotation (RL120) under initial level of N deposition (N4.9). The growth responses to increased N deposition were re-expressed as growth-response (ecophysiological) constraints on long-term productivity over a period of 120 years without rotation (RL120) under each increased deposition level (N18, N30, N50, N70 and N90). The model was then used to investigate the effects of rotation lengths (RL40, RL30, RL20, and RL15) on long-term forest productivity for initial (N4.9) or increased (N18, N30, N50, N70 and N90) N deposition. We examine the changes in long-term forest in response to different rotation length and increased N deposition, and figure out an appropriate rotation length for maximizing total stem biomass and average NPP. This approach did not address the issues of biological and genetic diversity conservation, or wider socioeconomic aspects of sustainability, such as energy costs associated with fertilizer inputs, harvest operations, transport and processing of wood products.

## Results

### Effects of Increased N Deposition

**Figure 2:**
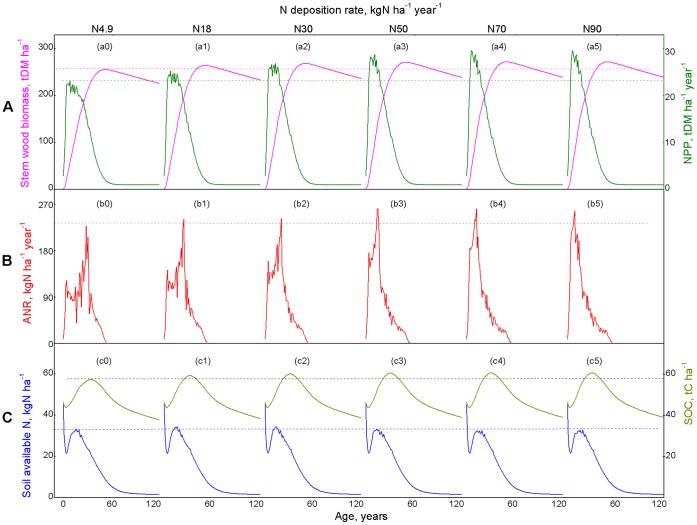
Simulated increased N deposition impacts on forest productivity as stand development. (Panel A) The magenta solid line stands for the stem wood biomass production in tone dry matter per hector (t DM ha^−1^), and green solid line refers to the net primary production in tone dry matter per hector (NPP, t DM ha^−1^ year^−1^). (Panel B) Annual increment of ANR (red solid line, ANR, kg N ha^−1^ year^−1^). (Panel C) The blue solid line represents current status of soil available N for growth in kg N per hector (kg N ha^−1^), olive solid line refers to soil organic C (SOC, t C ha^−1^) of *C. lanceolata* plantation in southern China from seedling to 120 years of age. In panels A, B, and C, a0, b0 and c0 represent growth trends from the basic, under initial level of N deposition (N4.9) and without rotation practice (RL120), simulation that made up the baseline data set; and a1–5, b1–5, as well as c1–5 represent predicted growth response trends generated under increased N deposition. The dashed lines represent growth constraint equilibrium state.

The simulated productivity and N dynamics (Panel A, B, C in Fig. 2) of a *C*. *lanceolata* plantation, in terms of stand growth under the baseline (N4.9 scenario; Fig. 2a0, b0, c0) and increased N deposition impacts (N18, N30, N50, N70 and N90 scenarios; Fig. 2a1–5, b1–5, c1–5) for a period of 120 years, are presented in Fig. 2.

The baseline simulation (Fig. 2a0, b0, c0) generated a realistic sigmoid function of age in the general pattern of stand growth in which the total dry matter of stem wood biomass continuously increased with stand development, peaked at 255.84 t DM ha^−1^ at a stand age of 53 years, followed by a gentle decline as the stands grew, and yielded at 226.35 t DM ha^−1^ at the end of 120-year period (Fig. 2a0). The increase in wood production with stand age was corresponding to the “growth expansion first and decline later” pattern of NPP (Fig. 2a0). The decline trends as stand grows after 53 years can be explained from ANR (<0 after 54 years) (Fig. 2b0). NPP initially exhibited a steep increase during the first 10 years, approximated a maximum rate (here 22.08 t DM ha^−1^ year^−1^) at 13 years, and then gradually declined with growth. Thereafter, NPP decreased to nearly 0 (here 0.3 t DM ha^−1^ year^−1^) at 60 years. Decreased nutrient availability and enhanced stomatal limitation were the major causes for NPP decline with stand age. Possible shifts in allocation to belowground components also may contribute to the apparent decline in productivity and biomass accumulation rate of stem wood. The average NPP over the 120-year period was 5.78 t ha^−1^ year^−1^ (Appendix S2).

Annual N requirement (ANR) demonstrated a drastically different pattern compared with production as the stand developed, formed an age-related “concave up beginning convex later” trends (Fig. 2b0). In terms of increases in foliage and fine root pools, ANR increased sharply from 9 kg N ha^−1^ year^−1^ at 2 years to a maximum value of 109.6 kg N ha^−1^ year^−1^ at 16 years. Then caused by self-thinning, ANR generally decreased between 16 and 23 years of age, corresponded to NPP fluctuates at this period. During the period from 23 to 29 years, ANR increased from 70.7 kg N ha^−1^ year^−1^ to a maximum of 227.9 kg N ha^−1^ year^−1^. After 29 years, ANR decreased and fell to <0 after 54 years, which indicates any increase in stand level respiration, hydraulic resistance, and perhaps plant tissue maturation as age grew. The average ANR over the 120-year period was 32.4 kg N ha^−1^ year^−1^(Appendix S2).

Soil available N (Fig. 2c0) decreased from 55.9 kg N ha^−1^ at the time when the seedlings were planted to 21.5 kg N ha^−1^ at a stand age of 5 years (Fig. 2c0). As the stand developed, soil available N increased to 32.6 kg N ha^−1^ during the period from 5 to 15 years. Thereafter, soil available N decreased to 1.5 kg N ha^−1^ at 120 years of age. Similar to soil available N, SOC (Fig. 2c0) varied over the first few years after harvest and then declined by about 10% for the next 5 years (Fig. 2c0). SOC dropped from 48.3 t C ha^−1^ at the time when the seedlings were planted to 43.6 t C ha^−1^ at a stand age of 5 years, and then returned to the baseline level by a stand age of 15 years. Then, SOC increased reaching a peak value of 57.4 t C ha^−1^ at an age of 35 years. Thereafter, SOC decreased to 37.8 t C ha^−1^ at 120 years.

Fig. 2 a1–5, b1–5, c1–5 show the variation of stem wood biomass (t DM ha^−1^), NPP (t DM ha^−1^ year^−1^), ANR (kg N ha^−1^ year^−1^), soil available N (kg N ha^−1^) and SOC (t C ha^−1^) with stand ages, predicted by the model under increased N deposition rate (without the influence of harvesting regimes) over the 120 year study period. The results showed that productivity of *C*. *lanceolata* plantation in response to increased N deposition was more related to age than N addition depending on the proportion and age of stand development. Increased N deposition has shown a rapid peak in growth and N dynamics. Increasing N depositions increased average NPP to 5.95, 6.032, 6.05, 6.06, and 6.06 t DM ha^−1^ year^−1^ under the N deposition rates of 18, 30, 50, 70 to 90 kg N ha^−1^ year^−1^, respectively (Appendix S2). Increased available N, decreased stomatal limitation and early arrival of a large leaf area were the major causes for the growth peaks decline with stand age.

### Effects of Rotation Lengths Under the N4.9 Scenario

**Figure 3:**
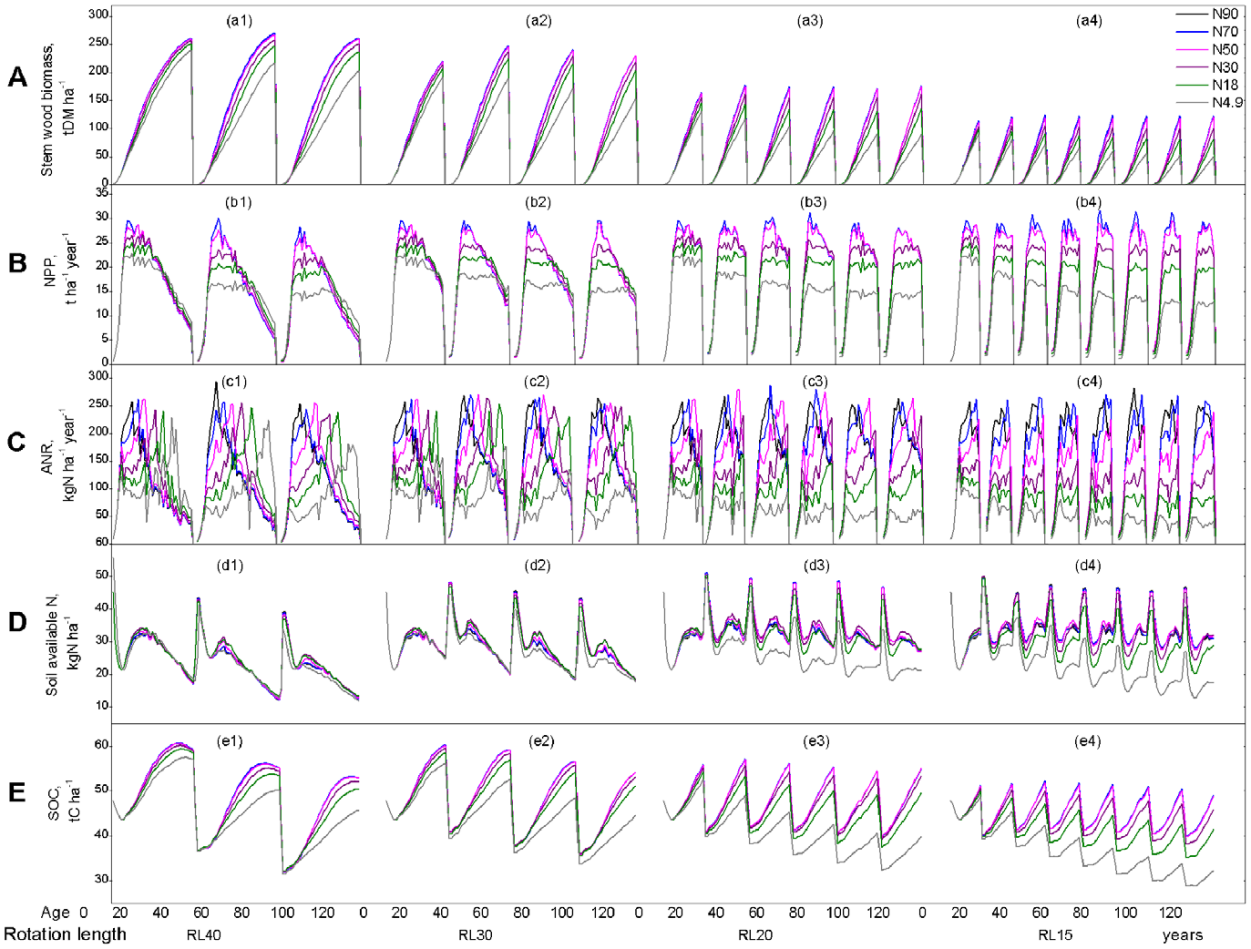
Simulated interactive effects of increased N deposition and short-rotation management as stand development. (A) The stem wood biomass production in tone dry matter per hector (t DM ha^−1^). (B) The net primary production in tone dry matter per hector (NPP, t ha^−1^ year^−1^). (C) Annual increment of ANR (kg N ha^−1^ year^−1^). (D) The current status of soil available N for growth in kg N per hector (soil available N, kg N ha^−1^). (E) Soil organic C (SOC, t C ha^−1^). Over the 120-year period coupling chronic levels of atmosphere N depositions of N4.9, N18, N30, N50, N70, and N90 with different rotation intervals (RL40, RL30, RL20, and RL15). RL40, RL30, RL20, and RL15 represent rotation cycles with intervals of 40, 30, 20, and15 years, respectively. N4.9 (black solid line) = 4.9 kg N ha^−1^ year^−1^, N18 (gray solid line) = 18 kg N ha^−1^ year^−1^, N30 (light gray solid line) = 30 kg N ha^−1^ year^−1^, N50 (black dash) = 50 kg N ha^−1^ year^−1^, N70 (gray dash) = 70 kg N ha^−1^ year^−1^, and N90 (light gray dash) = 90 kg N ha^−1^ year^−1^.

**Figure 4:**
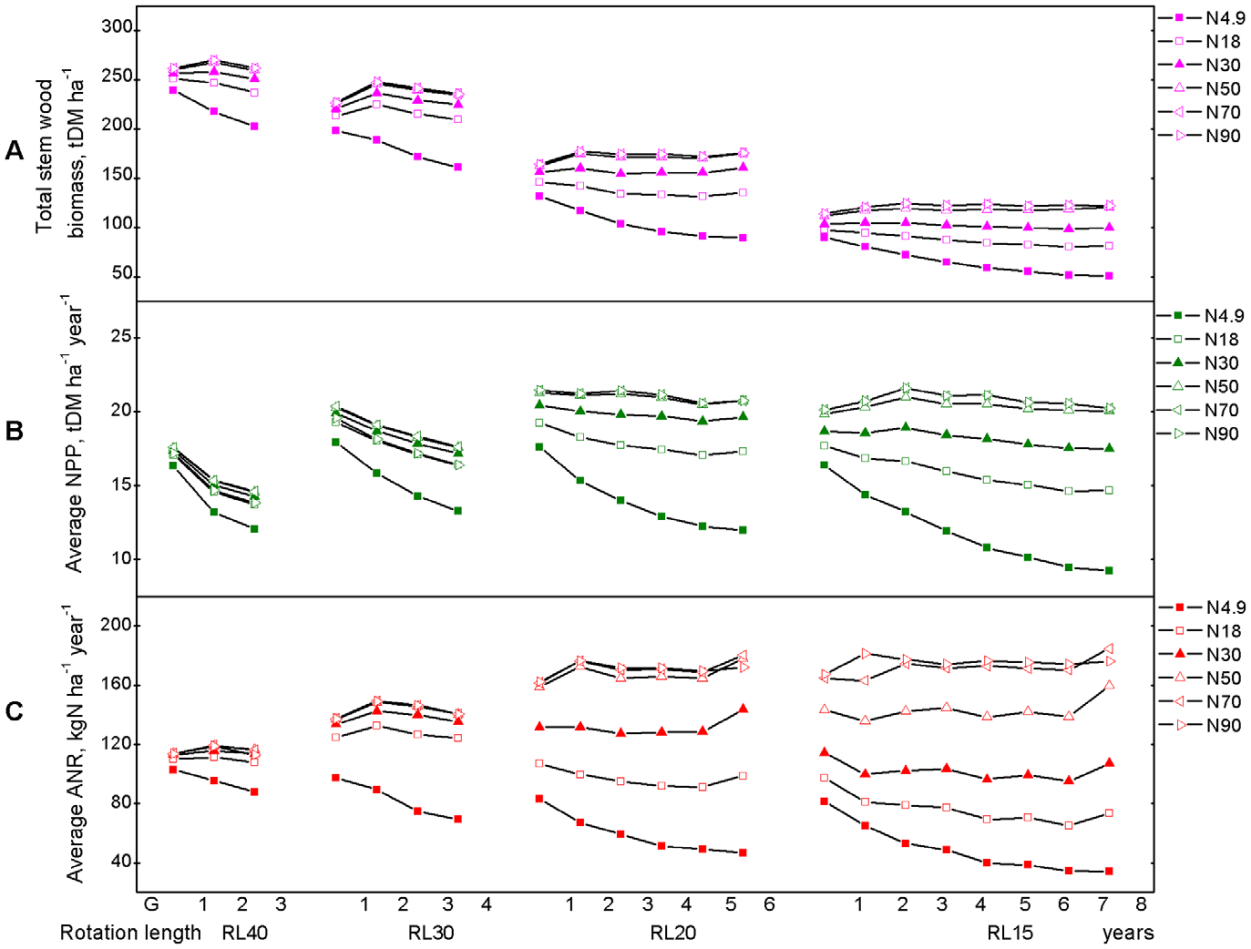
The summary of simulated interactive effects of increased N deposition and short-rotation management over successive rotations. It displays the accumulative statues, (A) Stem wood biomass at the end-of-rotation, and rotation-average values for flux or rates, (B) NPP and (C) ANR. RL40, RL30, RL20, and RL15 represent rotation cycles with intervals of 40, 30, 20, and 15 years, respectively. The effects of atmosphere N deposition levels: N4.9 (solid squares) = 4.9, N18 (hollow squares) = 18, N30 (solid upward triangles) = 30, N50 (hollow upward triangles) = 50, N70 (hollow leftward triangles) = 70, and N90 (hollow rightward triangles) = 90 kg N ha^−1^ year^−1^.

**Figure 5:**
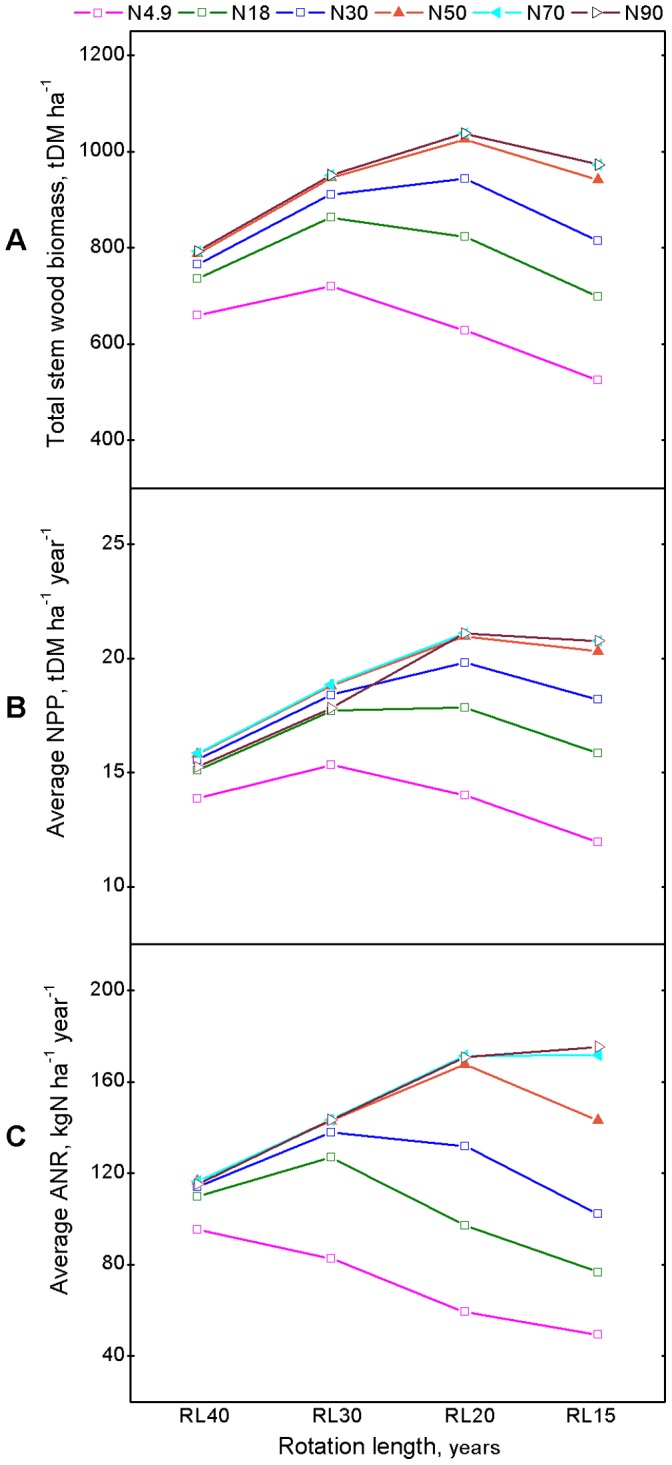
The long-term (total 120-year period) summary of interactive effects of increased N deposition and short-rotation management over successive rotations. It shows the sum of accumulative statues at the end-of-rotations, (A) stem wood biomass, and 120-year-average values for flux or rates, (B) NPP and (C) ANR.

Under the null level of N deposition (N4.9; gray solid lines in Fig. 3, solid squares in Fig. 4, and pink solid line with hollow squares in Fig. 5), the effects of rotation lengths on the long-term productivity of *C*. *lanceolata* plantation are shown in Figs. 3,4,5 and Appendix S2.

Over 120 year period, rotation length resulted in the changes in the productivity and N dynamic of *C*. *lanceolata* plantation with successive rotations (Fig. 3A, B, C, D, E). Soil available N decreased with the increase in successive rotations (Fig. 3D). First rotation plantation exhibited conservative N-cycling properties. As successive rotation practiced, N-cycling properties recovered with increasing availability of soil nitrate relative to ammonium. The dominance of a conservative N cycle for typical mature lowland tropical forests re-emerged [39]. All rotation lengths led to a yield decline in stem wood biomass accumulation (Fig. 3A), annual dry matter biomass increment (NPP, Fig. 3B), and annual increment of ANR (ANR, Fig. 3C) at harvest time over successive rotations. The shorter the rotation length, the greater the decrease in yields at harvest time (Figs. 3). For a certain rotation length (Fig. 4), the successive decrease was greatest for the first 1–3 rotations, usually by about 5–25%, and tended to be modest thereafter (Fig. 4). However, stem wood biomass demonstrated an increase during rotations 5–6 of the RL20 and 6–8 of the RL15 treatments, respectively (Fig. 4A). On the long-term (Fig. 5), in general, over the 120-year period, total productivity (total stem biomass and average NPP) demonstrated “increase first decrease later” patterns as rotation length shortened (Fig. 5A, B), while the trends of average ANR peaked at RL40 (Fig. 5C), and total stem wood biomass and average NPP peaked at RL30 (Fig. 5A, B). Differences in total production between RL30, RL20, and RL15 gradually increased as the rotation length shortened, and this result was particularly evident in terms of the timber harvest. A rotation length of 30 years was optimal for sustainable management at the N4.9 deposition level.

### Interactive Effects of Rotation Length and Increased N Deposition

Rotation length clearly affected the productivity and N dynamics of the simulated *C*. *lanceolata* plantation over the 120-year period from perspective of growth response constraints (Figs. 3), successive effects (Fig. 4, Appendix S2), and long-term outcomes (Fig. 5, Appendix S2).

For different rotation lengths (RL40, RL30, RL20, and RL15) and the simulated productivity and N dynamic values of the *C*. *lanceolata* plantation over the 120-year cultivation period are presented in Fig. 3, 4 and 5. In general, on the long-term (Fig. 5), short rotation length had a more significant effect on productivity and N dynamics than high N deposition. Under all rotation length cycles, increasing N deposition generally led to increases in both productivity and N dynamics. However, the sensitivity of N deposition for a given rotation length decreased with increasing amounts of N deposition. Over the 120-year period, the total stem biomass, average NPP, and average ANR did not sensitively response to increase in N deposition level higher than N50 (50 kg N ha^−1^ year^−1^). This result indicated that a N deposition level of 50 kg N ha^−1^ year^−1^ was the upper limit level of thresholds for N deposition impacts for *C*. *lanceolata* plantations with rotation length of 30 and 40 years (Fig. 5). Similarly, N70 (70 kg N ha^−1^ year^−1^) was the upper limit level of thresholds for N deposition impacts under a rotation length of 15 and 20 years (Fig. 5).

For all of the increased N deposition scenarios larger than 50 kg N ha^−1^ year^−1^, all indicators increased as the rotation length was shortened from RL40 to RL20 over the entire 120-year simulated period and exhibited moderate variations in average ANR when the rotation length was changed from RL20 to RL15 (Fig. 5). These results indicate that the largest increase in total stem wood biomass and average NPP occurred when using the RL20 cycle and smaller differences in total stem wood biomass and average NPP occurred between RL15 and RL30 (Fig. 5A, B). At a specific N deposition level, over the 120-year period, a rotation length of 20–30 years would be the most sustainable practice in terms of total stem wood biomass, average NPP and average ANR under the N18 scenario (Fig. 5A, B, C), while under the N30 scenario, a rotation length of 20 years would be most sustainable in terms of total stem wood biomass and average NPP (Fig. 5A, B), and for N50, N70 and N90 scenarios, the sustainable rotation length would be 15–20 years in terms of total stem wood biomass and average NPP (Fig. 5A, B).

At the same time, a shorter rotation length resulted in a major decline in forest floor biomass. A change in the rotation length from RL40 to RL30 increased SOC by 0.15% under N18, 0.18% under N30, and 0.015% under N50, but decreased SOC by 15% under N70 and N90. Therefore, for N deposition levels higher than N18, rotation lengths <30 years lead to 2.5–8.2% losses in SOC (Fig. 3E).

## Discussion

### Uncertainties of Model Performance

The results of this study may be limited by four aspects of uncertainties: (1) resulted from complex interactions among the different components of the forest, varied in different forest regions, elevations, among different temporal and spatial scales by comprising multi-level of eco-biological complexity, the response of a forest ecosystem to climate change can have very different magnitudes and even different directions [40]–[42]. Available studies suggest that forest responses to climate change factors (such as increasing temperature and CO_2_) will be limited by competition, disturbance, and nutrient limitations [43]–[44]. (2) CenW model was unable to acquire sufficient field measurements of reference parameters from literature, pertaining to model parameterization as it relates to *C*. *lanceolata* stands, fixed parameters of default values or average values from literature or field measurements were used in this study (see Appendix S1). (3) Our study concentrated on how N deposition and climate would affect the growth for *C*. *lanceolata* plantation. As mentioned before, we didn’t consider land use change resulted from human activities, disturbances such as fires and environmental pollution as aerosols, atmospheric CO_2_ and O_3_, which are important factors affecting forest productivity and N dynamics [45]–[46]. (4) Although our results agree with many other studies at realistic temporal scales, but long-term productivity modeling still has limited knowledge of the forest responses to increased N deposition in the field on the long-term observation.

### Model Simulation of Effects of Increased N Deposition

The simulated NPP of the *C*. *lanceolata* plantation demonstrated a steep increase during the first 10 years, reaching its maximum growth rate at the age of 13 years, which then declined thereafter. This growth pattern is in good agreement with the measured peak NPP values of evergreen coniferous forests in tropical and subtropical zones [47]. The simulated average NPP values during the first 60 years under the N4.9 scenario (4.9 kg N ha^−1^ year^−1^) and RL120 length (11.34 t DM ha^−1^ year^−1^) in this study are within the range of the average NPP (9.02–15.47 t DM ha^−1^ year^−1^) measured in *C*. *lanceolata* plantations at the same site [48]. These results are close to those values measured in subtropical coniferous forests in China [49], [50]. Over the simulated 120-year period, the simulated average NPP (5.78 t DM ha^−1^ year^−1^) is within the range of values measured in the forests of China (approximately 4.8–6.22 t DM ha^−1^ year^−1^) [51], indicating that *C*. *lanceolata* is a fast growing tree species with a high production value. Despite some uncertainties existed in this study, the simulated results for initial N deposition level (N4.9) could be explained by the five factors reviewed by Ryan et al. [52], including changes in photosynthesis rate as stand development, nutrient supply, respiration, C allocation, and hydrological function. Our study also implied that a decline in stand leaf area usually accompanies a decline in aboveground wood growth. The decline in NPP with stand age can be explained as a combined effect of declining photosynthesis efficiency and declining N-availability for tree growth [53].

The results showed that of the productivity of *C*. *lanceolata* plantation in response to increased N deposition were more related to stand age than N addition depending on the proportion and age of growing forests. Increased N deposition has shown to raise the equilibrium state and constraint curve. Increased rates of N deposition can affect site nutrient stores and dynamics, microclimate, and tree growth. Recent evidence suggests that sustained growth response is unlikely to be realized as a cumulative effect of N fertilization, but such a possibility might exist with other forms of fertilization, if they become widespread [54]. Based on the assumption that duration of these effects is short, cumulative nutrient mineralization and/or leaching should be unlikely to occur as cumulative effects over time on single site. However, duplication of applications across landscape areas might hypothetically lead to cumulative increases in “baseline” nutrient mobilization and/or leaching for the area as a whole.

### Interactive Effects of Increased N Deposition and Short-rotation Length

Maintaining long-term productivity in forests is a fundamental goal of ecologically sound forest management [55]. Factors that may be detrimental to the sustainability of plantations include the removal of a large proportion of material from the site, short-rotation length, and increased site disturbances [18]. Of particular concern is the sustainability of the physical properties of the soil, soil horizons, and complex biogeochemical cycles that are constantly at work within the forest system [28]. A number of studies have investigated the effects of harvesting regimes on the rotation cycle, growth rate, and C storage [18], [28], [32], [56]. Conventional stem-only harvesting is accepted as a sustainable forestry practice [57].

In this study, rotation length was an important factor that affects long-term productivity and N dynamics of *C*. *lanceolata* plantations if the N deposition level is low. As expected, total productivity over the 120-year simulation period demonstrated a nonlinear response pattern: specifically, an initial increase in productivity, which declined in accordance with a shorter rotation length. The highest value (15.25 t DM ha^−1^ year^−1^) was achieved using a 30-year rotation cycle (RL30) (Fig. 5B). Therefore, under current climatic conditions and an N deposition level of 4.9 kg N ha^−1^ year^−1^ (N4.9), a reasonable rotation cycle was determined to be 30 years in terms of sustainable productivity over the 120-year simulated period (Fig. 5B).

The rotation length also strongly affects the productivity and N dynamics over successive rotations at *C*. *lanceolata* plantations, which mostly monocultures that are currently managed using rotation lengths <20 years [28]. Short rotation cycles and intensive forest practices in the past have been linked to yield declines, soil depletion, and reductions in productivity over successive rotations in *C*. *lanceolata* plantations [4], [28], [30]. Significant declines in the yield (10–40%) have been reported [25], [27]. In this study, the simulated soil available N was highest in the second rotation for rotation lengths <40 years (RL40) (Fig. 3D). The simulated stem wood biomass, NPP, ANR, and SOC values were highest during the first rotation regardless of the rotation cycle, then, decreased as the number of rotation generations increased (Fig. 3A, B, C, E). The decreasing rate of SOC varied over successive rotations, demonstrating a sharp decrease by about 5–25% over the first 1–3 generations, but only a modest successive decrease was demonstrated thereafter. However stem wood biomass increased after the sixth RL20 rotation and the eighth RL15 rotation (Fig. 4A). The decline in yield over successive rotations, as observed in the model simulation, is consistent with the results of Zhang et al. [30], who reported that increments in the stand biomass were reduced by an average of 24% from the first to the second rotation and by a further 40% from the second to the third rotation. SOC was reduced by 10% between the first and the second rotations and by 15% between the second and the third rotations.

Changes in the composition and biomass of forest floors, associated with decomposition, nutrient mobilization, and redistribution from vegetation and forest floors to mineral soil horizons should occur commonly as a cumulative effect in managed forests, as these trends are influenced in similar or complementary ways by several forestry practices, including harvest, site preparation, fertilization and herbicide application. The long-term significance of these trends is unknown. Short-rotation management aimed to increase wood production may affect forest sustainability depending, among other factors, on initial site conditions and treatment regimes. Excessive removal of C and nutrients and soil disturbance are two of the major factors that may cause site degradation and reduction in forest productivity [58]. Short-rotation practices can increase productivity above base level and reduce or eliminate negative impacts. Currently, there is a little direct evidence to support that harvest removals in themselves lead to soil depletion over several succeeding rotations. Short-term productivity declines should be due to severe associated effects such as compaction, erosion or loss of organic layers.

The simulation results of this study indicate that the rotation length and N deposition level interactively affect the productivity and N dynamics of *C*. *lanceolata* plantations. Even though rotation length is an important factor that affects the productivity and N dynamics of *C*. *lanceolata* plantations, the offsite effects of the shorter rotation length decreased as the amount of N deposition increased. A high level of N deposition led to increased production and N accumulation in the plantation ecosystem, but the increasing effects of production due to high N deposition increased as the rotation length was shortened. These phenomena could be interpreted by the fact that high N deposition surpassed the loss of N in the soil due to the removal of harvesting materials and leaching due to runoff.

Continuously increasing atmospheric N deposition will result in soil acidification, N leaching, nutrient imbalances, and NPP depression in forests [4], [59]. The results of this study imply that the offsite detrimental effects of chronic atmospheric N deposition could be ameliorated through appropriate forest management initiatives such as rotation length selection. For example, for a given level of N deposition, the responses of NPP to a high level of N deposition were strongly correlated with stand age (Fig. 2). Over the 120-year period, the total stem biomass, average NPP, and average ANR did not sensitively response to increase in N deposition level higher than N50 (50 kg N ha^−1^ year^−1^). This result indicated that N deposition level of 50 kg N ha^−1^ year^−1^ was the upper limit level of thresholds for nitrogen deposition impacts for *C*. *lanceolata* plantations with as rotation length of 30 and 40 years (Fig. 5). Similarly, N70 (70 kg N ha^−1^ year^−1^) was the upper limit level of thresholds for nitrogen deposition impacts under a rotation length of 15 and 20 years (Fig. 5). The NPP sensitive to the N deposition level of 18–50 kg N ha^−1^ year^−1^ (N18, N30 and N50) occurred when the stand age was 6–20 years, while the sensitive of NPP to high N deposition, such as N70 (70 kg N ha^−1^ year^−1^) or N90 (90 kg N ha^−1^ year^−1^), occurred when the stand age was 6–19 years. Therefore, the sensitive stand age of N increasing was around 20 years for atmospheric N deposition >18 kg N ha^−1^ year^−1^ input to the *C*. *lanceolata* plantation. After the stand age of N sensitive, N deposition would exceed N uptake by the trees and the retention capacity, consequently resulting in negative environmental impacts. The sensitive stand age of N increasing for a specific N deposition level may be the appropriate rotation length to sustain productivity without offsite environmental effects in the *C*. *lanceolata* plantation.

The results of this study also indicate that increasing atmospheric N deposition might promote improvements in tree growth and site quality at *C*. *lanceolata* plantations. While very short rotation lengths reduced the capacity of the plantation ecosystem to accumulate C and N, particularly in sites with initially poor levels of nutrients, these reductions could have been averted by higher atmospheric N deposition. The obvious positive responses in terms of productivity and reduced N-use efficiency in *C*. *lanceolata* plantations due to increasing N deposition are consistent with another study on *C*. *lanceolata* plantations in southeastern China, which simulated results using the FORECAST model [32]. For example, increasing N deposition from 4.9 kg N kg N ha^−1^ year^−1^ (N4.9) to 70 kg N ha^−1^ year^−1^ (N70) resulted in higher productivity and timber biomass, increased forest N retention, and increased N requirements for tree growth, but lower SOC accumulation and decreased soil available N in *C*. *lanceolata* plantations. Further increasing N deposition to N90 (90 kg N ha^−1^ year^−1^) or higher did not increase productivity, N retention, or affect SOC or soil available N. Therefore, N deposition>N70 (70 kg N ha^−1^ year^−1^) leads to the loss of N through leaching and offsite effects.

### Conclusions

Case studies that use modeling are needed to improve our understanding of the effects of the rotation cycle and N deposition on the long-term productivity of plantations. In terms of how the model simulations performed when they incorporated variables from a *C*. *lanceolata* plantation in subtropical China, our results show that CenW3.1 demonstrated considerable potential for this particular application. Not only can the model be used to integrate data from various forest studies in a dynamic manner, thus enabling a more thorough examination of study data, it can also be applied to predict the potential long-term effects of management practices and N deposition on the functions (such as C and N processes) of a forest ecosystem. Over the long term, a combination of indicators, including NPP, stem wood biomass and ANR indicate that 20–30 years is a sustainable rotation cycle for subtropical *C*. *lanceolata* plantations assuming an N deposition level of 4.9–50 kg N ha^−1^ year^−1^; shorter rotation lengths (15–20 years) may be sustainable for N deposition rates between 50 and 70 kg N ha^−1^ year^−1^, to avoid leaching and offsite effects.

## Supporting Information

Appendix S1 Definitions, symbols, values, units, and sources of parameters used in CenW3.1 modeling of a *C*. *lanceolata* plantation. For the “Value sources” column, [8], [23], [31], [37], [65]–[72] are references citations, D = default, F = fitted, O = observed, and A = assumed.
(DOC)

Appendix S2 The summarized values for each rotation and over 120-year period (All) in *C*. *lanceolata* plantation under different N deposition levels (N4.9, N18, N30, N50, N70 and N90 for 4.9, 18, 30, 50, 70, and 90 kg N ha^−1^ year^−1^) and rotational lengths (RL120, RL40, RL30, RL20 and RL15 for no harvesting, 40, 30, 20, and 15-year rotation).
(DOC)
