# Supplementary material for: Effects of Increased Nitrogen Deposition and Rotation Length on Long-Term Productivity of Cunninghamia lanceolata Plantation in Southern China
Source: PLoS One. 2013 Feb 4;8(2):e55376. doi: 10.1371/journal.pone.0055376 (PMC3563596; doi:10.1371/journal.pone.0055376)
Supplement: Appendix S1 — Definitions, symbols, values, units, and sources of parameters used in CenW3.1 modeling of a C. lanceolata plantation. For the “Value sources” column, [8], [23], [31], [37], [65]–[72] are references citations, D = default, F = fitted, O = observed, and A = assumed. (DOC) [file pone.0055376.s001.doc]

**Appendix S1** Definitions, symbols, values, units, and sources of parameters used in CenW3.1 modeling of a *C*. *lanceolata* plantation. For the “Value sources” column, [8, 23, 31, 37, 65-72] are references citations, D = default, F = fitted, O = observed, and A = assumed.

| Description | Units | Value for *C*. *lanceolata* plantation | Value sources |
| --- | --- | --- | --- |
| Site parameter |  |  |  |
| System nutrient dynamics |  |  |  |
| Volatilization fraction (N) | - | 0.01 | F |
| Atmospheric input (N) | kg ha^-1^ yr^-1^ | 4.9 | [37] |
|  |  | 18 | [8] |
|  |  | 30, 50, 70, 90 | A |
| Leaching fraction (N) | - | 0.148 | [64] |
| Daily release rate of applied fertilizer | - | 0.1 | D |
| Maximum rate of soil evaporation | - | 0.18 | [65] |
| Water-holding capacity of litter | g gDW^-1^ | 2.745 | [66] |
| Mulching effect on water loss | % tDW^-1^ | 5 | O |
| Canopy aerodynamic resistance | s m^-1^ | 2.94 | [67] |
| Warm melt | mm def^-1^ d^-1^ | 0.8 | D |
| Radn (radiation) melt | mm (MJ m^-2^)^-1^ | 0.2 | D |
| Resistance to soil T change | - | 5 | D |
| Snow insulation | r mm^-1^ | 10 | D |
| Max. rate of soil evap. | - | 0.18 | D |
| Soil insulation effects of LAI | - | 0.5 | D |
| Direct evaporation calculation |  |  |  |
| Slop of relationship relating water interception of foliage to LAI, when calculated from "Amount intercepted=f(LAI)" | - | 4.32 | [68] |
| Fraction of rain lost to direct evaporation, when calculated from "Constant fraction of rain" | - | 0.2 | [69] |
| Stand Parameters |  |  |  |
| Mortality |  |  |  |
| Ratio dieing (average trees) | - | 0.2 | F |
| Mortality fraction | - | 0.01 | F |
| Parameter in 3/2 power law | - | 15.9 | F |
| Annual senescence |  |  |  |
| Foliage minimum | - | 0.25 | O |
| Bark | - | 0.3 | O |
| Branches | - | 0.2 | O |
| Fruit | - | 0.5 | O |
| Fine roots | - | 1 | O |
| Pollen | - | 1 | O |
| Longevity of sapwood | y | 15 | O |
| Foliage senescence in dense canopy |  |  |  |
| Low-light limit | MJ m^-2^ d^-1^ | 0.1 | O |
| Max daily senescence |  | 0.003 | O |
| Use of carbohydrates and soluble N |  |  |  |
| Carbohydrate K_m_ as % of live tissue | - | 50 | O |
| Soluble N K_m_ as % of live tissue | - | 62.3 | O |
| Ratio of average N in foliage and N in top layer | - | 0.811 | [65] |
| Ratio of N in senescing and living foliage | - | 0.824 | [65] |
| Wood density | kg m^-3^ | 0.44 | [65] |
| Fraction of soil microbial N that is directly taken up | g d^-1^ kg^-1^ | 0.0238 | [37] |
| Amount of N biologically fixed per unit of fixed C | gN kgC^-1^ | 0.1528 | O |
| Maximum daily death rate of foliage during drought | % d^-1^ | 0.01 | O |
| Water Stress Limit (Relative soil water content that starts plant water stress) | - | 0.3 | [70] |
| Respiration ratio | - | 0.5 | F |
| Photosynthetic terms |  |  |  |
| Specific leaf area | m^2^ kg^-1^ | 5.9918 | [71] |
| Foliage albedo |  | 0.125 | [72] |
| Transmissivity |  | 0.01 | [72] |
| Loss as NMVOC |  | 0.01 |  |
| Leaf photosynthetic parameters (top of canopy) |  |  |  |
| No(the foliar nitrogen concentration at which photosynthesis is 0) | g kg^-1^ | 5 | D |
| Saturating N | g kg^-1^ | 20 | O |
| Max N | g kg^-1^ | 30 | O |
| Max A with CO_2_ non-limiting (Maximum Assimilation Rate) |  | 35 | O |
| Maximum quantum yield |  | 0.1 | [31] |
| Curvature of light response |  | 0.7 | F |
| Maximum light extinction coefficient |  | 0.5 | D |
| Stomatal conductance parameters |  |  |  |
| Unstressed |  | 16.4 | D |
| Stressed |  | 8.2 | D |
| Temperature damage parameters |  |  |  |
| TFrost (It is assumed that plants could be damaged by either cold (frost) or heat (scorch). |  | 0 | O |
| Tscorch |  | 40 | O |
| Sensitivity |  | 0.05 | O |
| Repair rate |  | 2 | O |
| Maximum length for complete repair |  | 5 | O |
| Age- or size-related photosynthetic decline |  |  |  |
| Stand maturity | tDM ha^-1^ yr^-1^ | 300; 40 | [23] |
| Power term |  | 4 | [31] |
| Allocation |  |  |  |
| Carbon allocation ratios |  |  |  |
| Fine root:foliage |  | 0.302 (unstressed); 1.8 (stressed) | [65] |
| Foliage: branch (H = 10 m) |  | 1.15 | [65] |
| Stemwood:branch |  | 2.5 | [65] |
| Coarse roots:stemwood |  | 0.179 | [65] |
| Bark:stemwood |  | 0.152 | [65] |
| Allocation to fruits |  | 0.0047 | [65] |
| Allocation to pollen |  | 0.0001 | [65] |
| Minimum age for reproduction | y | 10 | [65] |
| Excess N uptake ratio |  | 0.8 | [65] |
| Minimum allocation to stemwood |  | 0.4 | [65] |
| Ration of N in heartwood and sapwood |  | 0.25 | [65] |
| Stem allometric relationships (Wt=f(D, H)) |  |  |  |
| DBH |  | 1.694 | F |
| H |  | 0.847 | F |
| Allometric relationship height vs DBH |  |  |  |
| Intercept |  | 0.189 | F |
| Slope |  | 0.83 | F |
| Min dbh for allom.eqn. | cm | 4.0 | O |
| Ratio of N in plant component:N in foliage |  |  |  |
| Stemwood:branch |  | 0.057 | F |
| Bark:stemwood |  | 0.228 | F |
| Fine roots |  | 0.325 | F |
| Branches |  | 0.463 | F |
| Fruits |  | 0.4 | F |
| Pollen |  | 0.8 | F |
| Weather Parameters |  |  |  |
| CO_2_ concentration |  | 380 | O |
| Atmospheric pressure |  | 1000 | O |
