# Supplementary material for: Effects of Increased Nitrogen Deposition and Rotation Length on Long-Term Productivity of Cunninghamia lanceolata Plantation in Southern China
Source: PLoS One. 2013 Feb 4;8(2):e55376. doi: 10.1371/journal.pone.0055376 (PMC3563596; doi:10.1371/journal.pone.0055376)
Supplement: Appendix S2 — The summarized values for each rotation and over 120-year period (All) in C. lanceolata plantation under different N deposition levels (N4.9, N18, N30, N50, N70 and N90 for 4.9, 18, 30, 50, 70, and 90 kg N ha−1 year−1) and rotational lengths (RL120, RL40, RL30, RL20 and RL15 for no harvesting, 40, 30, 20, and 15-year rotation). (DOC) [file pone.0055376.s002.doc]

**Appendix S2 The summarized values for each rotation and over 120-year period (All) in *C*.*lanceolata* plantation under different N deposition levels (N4.9, N18, N30, N50, N70 and N90 for 4.9, 18, 30, 50, 70, and 90 kg N ha^-1^ year^-1^) and rotational lengths (RL120, RL40, RL30, RL20 and RL15 for no harvesting, 40, 30, 20, and 15-year rotation).**

| N deposition level, kg N ha^-1^ year^-1^ | Rotation length, year | Rotation | Stem wood biomass, t DM ha^-1^ | NPP, t DM ha^-1^year^-1^ | ANR, kg N ha^-1^ year^-1^ |
| --- | --- | --- | --- | --- | --- |
| N4.9 |  |  |  |  |  |
|  | RL120 | 1 | 226.35 | 5.78 | 32.37 |
|  |  | ALL | 226.35 | 5.78 | 32.37 |
|  |  |  |  |  |  |
|  | RL40 | 1 | 239.43 | 16.07 | 101.74 |
|  |  | 2 | 217.74 | 13.01 | 95.02 |
|  |  | 3 | 205.55 | 11.96 | 86.50 |
|  |  | ALL | 537.76 | 12.14 | 94.42 |
|  |  |  |  |  |  |
|  | RL30 | 1 | 198.23 | 17.73 | 101.82 |
|  |  | 2 | 189.08 | 15.69 | 92.085 |
|  |  | 3 | 171.56 | 14.27 | 76.01 |
|  |  | 4 | 166.89 | 13.31 | 70.80 |
|  |  | ALL | 642.21 | 14.13 | 85.18 |
|  |  |  |  |  |  |
|  | RL20 | 1 | 139.69 | 17.74 | 83.52 |
|  |  | 2 | 117.14 | 15.50 | 66.89 |
|  |  | 3 | 103.55 | 13.93 | 59.76 |
|  |  | 4 | 95.70 | 12.98 | 51.46 |
|  |  | 5 | 91.01 | 12.43 | 47.96 |
|  |  | 6 | 95.12 | 12.19 | 46.87 |
|  |  | ALL | 725.77 | 15.25 | 59.41 |
|  |  |  |  |  |  |
|  | RL15 | 1 | 98.68 | 16.77 | 67.10 |
|  |  | 2 | 80.25 | 14.61 | 53.50 |
|  |  | 3 | 72.33 | 13.25 | 48.87 |
|  |  | 4 | 65.08 | 11.92 | 40.38 |
|  |  | 5 | 58.91 | 10.93 | 38.80 |
|  |  | 6 | 55.51 | 10.36 | 34.88 |
|  |  | 7 | 51.74 | 9.73 | 33.69 |
|  |  | 8 | 55.26 | 9.56 | 33.69 |
|  |  | ALL | 662.72 | 13.68 | 43.86 |
| N18 |  |  |  |  |  |
|  | RL120 | 1 | 233.65 | 5.95 | 33.88 |
|  |  | ALL | 233.65 | 5.95 | 33.88 |
|  |  |  |  |  |  |
|  | RL40 | 1 | 251.32 | 16.78 | 108.38 |
|  |  | 2 | 246.88 | 14.32 | 109.30 |
|  |  | 3 | 238.77 | 13.48 | 103.48 |
|  |  | ALL | 715.90 | 16.13 | 107.06 |
|  |  |  |  |  |  |
|  | RL30 | 1 | 213.21 | 19.0` | 124.39 |
|  |  | 2 | 224.48 | 17.77 | 132.56 |
|  |  | 3 | 215.40 | 16.95 | 127.03 |
|  |  | 4 | 215.10 | 16.28 | 122.52 |
|  |  | ALL | 840.34 | 17.99 | 126.63 |
|  |  |  |  |  |  |
|  | RL20 | 1 | 154.68 | 19.38 | 109.15 |
|  |  | 2 | 142.25 | 18.49 | 101.23 |
|  |  | 3 | 134.38 | 17.69 | 96.80 |
|  |  | 4 | 133.27 | 17.5` | 94.63 |
|  |  | 5 | 131.48 | 17.28 | 92.37 |
|  |  | 6 | 144.27 | 17.56 | 96.58 |
|  |  | ALL | 868.19 | 17.50 | 98.46 |
|  |  |  |  |  |  |
|  | RL15 | 1 | 107.08 | 18.14 | 99.80 |
|  |  | 2 | 94.29 | 17.15 | 90.87 |
|  |  | 3 | 91.11 | 16.69 | 80.62 |
|  |  | 4 | 87.29 | 15.96 | 78.32 |
|  |  | 5 | 84.20 | 15.60 | 71.66 |
|  |  | 6 | 82.64 | 15.36 | 71.77 |
|  |  | 7 | 80.26 | 15.01 | 68.02 |
|  |  | 8 | 89.01 | 15.12 | 70.14 |
|  |  | ALL | 736.98 | 14.86 | 78.90 |
| N30 |  |  |  |  |  |
|  | RL120 | 1 | 236.91 | 6.015 | 34.54 |
|  |  | ALL | 236.91 | 6.015 | 34.54 |
|  |  |  |  |  |  |
|  | RL40 | 1 | 256.82 | 17.06 | 111.01 |
|  |  | 2 | 257.79 | 14.74 | 113.59 |
|  |  | 3 | 252.28 | 14.03 | 108.66 |
|  |  | ALL | 834.82 | 18.51 | 111.08 |
|  |  |  |  |  |  |
|  | RL30 | 1 | 220.59 | 19.58 | 131.69 |
|  |  | 2 | 236.51 | 18.37 | 141.12 |
|  |  | 3 | 228.96 | 17.62 | 137.89 |
|  |  | 4 | 229.66 | 17.00 | 133.39 |
|  |  | ALL | 959.73 | 19.89 | 136.02 |
|  |  |  |  |  |  |
|  | RL20 | 1 | 163.41 | 20.52 | 135.51 |
|  |  | 2 | 160.38 | 20.17 | 135.83 |
|  |  | 3 | 154.80 | 19.69 | 130.86 |
|  |  | 4 | 155.71 | 19.66 | 133.71 |
|  |  | 5 | 155.51 | 19.51 | 133.30 |
|  |  | 6 | 169.92 | 19.78 | 142.26 |
|  |  | ALL | 915.72 | 18.14 | 135.24 |
|  |  |  |  |  |  |
|  | RL15 | 1 | 114.60 | 19.22 | 118.49 |
|  |  | 2 | 104.62 | 18.89 | 113.20 |
|  |  | 3 | 104.64 | 18.97 | 106.26 |
|  |  | 4 | 102.06 | 18.43 | 105.50 |
|  |  | 5 | 100.92 | 18.40 | 101.04 |
|  |  | 6 | 99.57 | 18.15 | 101.88 |
|  |  | 7 | 98.79 | 18.01 | 100.32 |
|  |  | 8 | 109.62 | 18.04 | 103.31 |
|  |  | ALL | 766.89 | 15.28 | 106.25 |
| N50 |  |  |  |  |  |
|  | RL120 | 1 | 239.11 | 6.05 | 34.90 |
|  |  | ALL | 239.11 | 6.05 | 34.90 |
|  |  |  |  |  |  |
|  | RL40 | 1 | 260.45 | 17.24 | 111.92 |
|  |  | 2 | 267.18 | 15.01 | 116.62 |
|  |  | 3 | 261.30 | 14.30 | 111.43 |
|  |  | ALL | 963.98 | 20.59 | 113.32 |
|  |  |  |  |  |  |
|  | RL30 | 1 | 225.45 | 19.93 | 134.66 |
|  |  | 2 | 245.74 | 18.74 | 146.33 |
|  |  | 3 | 239.24 | 18.01 | 143.14 |
|  |  | 4 | 239.37 | 17.38 | 137.77 |
|  |  | ALL | 1041.64 | 20.93 | 140.47 |
|  |  |  |  |  |  |
|  | RL20 | 1 | 169.48 | 21.28 | 155.90 |
|  |  | 2 | 174.91 | 21.19 | 171.98 |
|  |  | 3 | 171.24 | 20.97 | 164.90 |
|  |  | 4 | 171.45 | 20.82 | 165.67 |
|  |  | 5 | 170.22 | 20.53 | 164.03 |
|  |  | 6 | 184.34 | 20.78 | 167.73 |
|  |  | ALL | 949.79 | 18.51 | 165.03 |
|  |  |  |  |  |  |
|  | RL15 | 1 | 123.42 | 20.34 | 151.02 |
|  |  | 2 | 117.43 | 20.61 | 156.44 |
|  |  | 3 | 119.48 | 21.00 | 149.92 |
|  |  | 4 | 117.57 | 20.51 | 149.12 |
|  |  | 5 | 118.22 | 20.71 | 146.58 |
|  |  | 6 | 117.85 | 20.52 | 147.46 |
|  |  | 7 | 118.45 | 20.50 | 147.50 |
|  |  | 8 | 131.56 | 20.54 | 155.39 |
|  |  | ALL | 788.93 | 15.52 | 150.43 |
| N70 |  |  |  |  |  |
|  | RL120 | 1 | 239.64 | 6.06 | 34.99 |
|  |  | ALL | 239.64 | 6.06 | 34.99 |
|  |  |  |  |  |  |
|  | RL40 | 1 | 261.34 | 17.28 | 111.75 |
|  |  | 2 | 269.90 | 15.06 | 116.94 |
|  |  | 3 | 262.95 | 14.32 | 110.96 |
|  |  | ALL | 995.39 | 20.99 | 113.22 |
|  |  |  |  |  |  |
|  | RL30 | 1 | 226.68 | 20.01 | 134.95 |
|  |  | 2 | 247.46 | 18.77 | 146.95 |
|  |  | 3 | 241.18 | 18.05 | 143.72 |
|  |  | 4 | 240.02 | 17.39 | 137.65 |
|  |  | ALL | 1053.82 | 21.04 | 140.82 |
|  |  |  |  |  |  |
|  | RL20 | 1 | 171.02 | 21.46 | 158.00 |
|  |  | 2 | 177.18 | 21.28 | 174.86 |
|  |  | 3 | 174.64 | 21.17 | 170.10 |
|  |  | 4 | 174.44 | 20.98 | 169.70 |
|  |  | 5 | 171.93 | 20.61 | 167.06 |
|  |  | 6 | 184.61 | 20.77 | 169.28 |
|  |  | ALL | 955.33 | 18.56 | 168.16 |
|  |  |  |  |  |  |
|  | RL15 | 1 | 125.75 | 20.63 | 167.58 |
|  |  | 2 | 120.69 | 20.97 | 179.36 |
|  |  | 3 | 124.62 | 21.55 | 175.21 |
|  |  | 4 | 122.41 | 21.00 | 172.44 |
|  |  | 5 | 123.77 | 21.24 | 173.12 |
|  |  | 6 | 122.06 | 20.92 | 171.31 |
|  |  | 7 | 122.71 | 20.91 | 172.86 |
|  |  | 8 | 133.37 | 20.71 | 172.72 |
|  |  | ALL | 794.19 | 15.55 | 173.07 |
| N90 |  |  |  |  |  |
|  | RL120 | 1 | 239.64 | 6.06 | 34.99 |
|  |  | ALL | 239.64 | 6.06 | 34.99 |
|  |  |  |  |  |  |
|  | RL40 | 1 | 261.34 | 17.28 | 111.54 |
|  |  | 2 | 269.90 | 15.06 | 116.56 |
|  |  | 3 | 262.95 | 14.32 | 110.69 |
|  |  | ALL | 995.42 | 20.99 | 112.93 |
|  |  |  |  |  |  |
|  | RL30 | 1 | 226.68 | 20.01 | 134.87 |
|  |  | 2 | 247.46 | 18.77 | 146.81 |
|  |  | 3 | 241.18 | 18.05 | 143.68 |
|  |  | 4 | 240.02 | 17.39 | 137.72 |
|  |  | ALL | 1053.83 | 21.04 | 140.77 |
|  |  |  |  |  |  |
|  | RL20 | 1 | 171.02 | 21.46 | 158.49 |
|  |  | 2 | 177.18 | 21.28 | 174.80 |
|  |  | 3 | 174.64 | 21.17 | 171.00 |
|  |  | 4 | 174.44 | 20.98 | 170.42 |
|  |  | 5 | 171.93 | 20.61 | 167.86 |
|  |  | 6 | 184.61 | 20.77 | 169.84 |
|  |  | ALL | 955.33 | 18.56 | 168.74 |
|  |  |  |  |  |  |
|  | RL15 | 1 | 125.75 | 20.63 | 169.52 |
|  |  | 2 | 120.69 | 20.97 | 182.41 |
|  |  | 3 | 124.62 | 21.55 | 177.37 |
|  |  | 4 | 122.42 | 21.00 | 174.64 |
|  |  | 5 | 123.79 | 21.24 | 175.81 |
|  |  | 6 | 122.06 | 20.92 | 174.50 |
|  |  | 7 | 122.71 | 20.91 | 175.88 |
|  |  | 8 | 133.38 | 20.71 | 175.57 |
|  |  | ALL | 794.19 | 15.55 | 175.71 |
